# Supplementary material for: Segmentectomy for ground glass-dominant invasive lung cancer with tumour diameter of 2–3 cm: protocol for a single-arm, multicentre, phase III trial (ECTOP1012)
Source: BMJ Open. 2024 Jul 3;14(7):e087088. doi: 10.1136/bmjopen-2024-087088 (PMC11227815; doi:10.1136/bmjopen-2024-087088)
Supplement: Supplementary data [file bmjopen-2024-087088supp001.pdf]

筛选号：□□□

肺段切除术治疗 2-3cm 磨玻璃成分为主型浸润性肺癌的单臂、多中心、前瞻性、验证性 III 期临床试验

知 情 同 意 书

方案号：FUSCC2211

版本号：2.0

版本日期：2023 年 01 月 01 日

知情页

【前言】

由于您已经被诊断可能患有非小细胞肺癌，故在此邀请您参加一项临床研究。在您同意参加该项研究之前，请您仔细阅读这份知情同意书，并向您的医生或研究人员提出任何您需要了解的问题，直至得到满意的答复。如果您同意参加本研究，请您签署此知情同意书，并且保存该份经您和研究者双方签字的知情同意书副本。

【研究目的和背景】

本研究的目的是评估肺段切除术对 2-3cm 磨玻璃成分为主型肺癌的疗效。

肺段切除术因其能够保留更多的正常肺组织而越来越受到重视。一项来自日本的单臂研究纳入了长径≤2cm、实性成分占比（consolidation-to-tumor ratio，CTR）≤0.25 周围型肺癌，并对其行亚肺叶切除，结果表明该组患者的术后五年无复发生存率（recurrence-free survival，RFS）达到 99.7%，且没有发生局部复发。而另一项来自日本的随机对照研究则研究了在长径≤2cm、0.5<CTR≤1 周围型肺癌中肺段切除术和肺叶切除术的治疗效果，结果表明肺段切除术的术后生存不劣于肺叶切除术，因此肺段切除术应该是周围型小肺癌的首选治疗方式。来自美国的一项随机对照研究也探究了在不大于 2cm 肺癌中亚肺叶切除术和肺叶切除术的治疗效果，也证实了亚肺叶切除可用于治疗 T1aN0 且肿瘤≤2cm 的肺癌。

然而，目前针对 2<长径≤3cm、CTR≤0.5 肺癌的手术方式还没有定论。一项来自日本的单臂临床研究（JCOG1211）纳入了下列患者：（1）2<长径≤3cm、CTR≤0.5；（2）长径≤2cm、0.25<CTR≤0.5；（3）长径≤2cm、CTR≤0.25 且楔形无法完全切除。只要患者符合上述条件的任意一条，就可纳入该研究，纳入的患者均接受了肺段切除。虽然，该研究纳入了 2<长径≤3cm、CTR≤0.5 肺癌患者，但是这个临床研究还含有相当一部分影像学非浸润的患者，这部分患者的预后极佳，这可能是导致整体预后较好的原因。所以，目前有必要针对 2<长径≤3cm、CTR≤0.5 浸润性肺腺癌的最佳手术方式展开探究。

【研究设计和步骤】

本研究为单臂、多中心、前瞻性、验证性 III 期临床研究。目前针对 2<长径≤3cm、CTR≤0.5 浸润性肺腺癌的患者，临床可以实行肺段切除术，也可以实行肺叶切除术。但肺段切除术，可以保留更多的正常肺组织。本研究纳入的患者将全部接受肺段切除术。如果您符合入选条件并同意参加该项研究的话，我们会记录您术中及术后的病理结果以用于后续分析统计。

您参与的这个研究将包括下列步骤：

➢ **筛选及术前评估期**

如果您选择参加本次临床研究，您的医生或其研究团队成员将首先评价该研究是否适合您。为了确定这一情况，在入组前 2 周内您将进行一次全面的医学检查，包括：

Chen S, *et al. BMJ Open* 2024; 14:e087088. doi: 10.1136/bmjopen-2024-087088

- 病史： 您的医生会问一些有关您健康的问题以及最近您使用的药物或其他治疗情况。重要的是告诉医生所有您正在使用药物或其他治疗，包括任何您自行购买的药物。
- 体格检查： 您的医生将对您的身体进行检查，包括测量身高、体重、体温、呼吸频率、脉搏以及血压等。您的医生也将对您进行日常生活能力的评估。
- 如果已活检，您的医生将分析您的病理标本，以明确病理结果为非小细胞肺癌
- 影像学检查：您的医生将分析您的胸部 CT、头颅核磁共振、彩超、骨扫描或 PET/CT 等影像学检查结果，以明确您的肺腺癌为临床 T1N0M0，Ia 期。
- 您的医生将通过心电图了解您的心脏情况。
- 常规血液检查： 将用来检查您的血细胞情况、凝血情况、肿瘤标记物癌胚抗原、肾脏或肝脏等器官的工作情况。这些检查大约需要收集 2 大汤匙的血液。
- 尿液检查： 用来排除泌尿系感染和蛋白尿等异常情况。

如果这些检查的结果提示参加该临床研究可能使您的健康受到危险，您的医生将不会建议您进入本次研究，并同您讨论其他的治疗选择。但如果这些检查结果显示您适合该研究，您可以选择参加这一临床研究。

➢ **手术治疗期**

签署知情同意书后，我们将按计划为您进行肺段切除术。在术中，您的医生将记录肿瘤冰冻病理的情况，该检查不会额外增加手术时间和创伤。

➢ **术后随访期**

如果您已入组并顺利接受了肺段切除术，术后 1 个月内，您的医生将记录石蜡病理的信息，包括肿瘤病理类型及淋巴结转移情况。

术后 5 年内，您会接到专业医师的随访电话，询问您疾病相关情况及恢复情况。

【可能的风险与不适】

本研究所有数据均基于临床已有数据，不会对您早成额外的创伤，但肺癌切除手术本身可能存在风险，您的医生将在手术前向您仔细介绍手术和麻醉的相关风险。

【可能的受益】

本次研究的治疗方案并不改变目前的标准临床实践。但您的健康状况和临床资料将有专职研究者进行评估。在试验期间，您也可能不会从本研究中直接获得更多的好处，但是您所提供的宝贵的医学信息将为日后非小细胞性肺癌患者的治疗提供帮助。

【标本采集】

本研究不涉及额外的标本采集，所有检验及检查均为术前常规检查，术中标本的评估也不需要额外采集组织标本

Chen S, *et al. BMJ Open* 2024; 14:e087088. doi: 10.1136/bmjopen-2024-087088

【肿瘤组织样本和血液样本留存】

如果您选择参加本项研究，为了更好地了解非小细胞肺癌及其治疗方法，研究者将使用您手术后切除的肿瘤组织的一部分样本用于将来与非小细胞肺癌有关的探索性研究。

您的肿瘤组织或标本可能会在相应的实验室或运送至中心实验室长期保存，直至样本全部用完或当时保存条件的极限。

被保存的您的肿瘤组织以及血标本的探索性研究可能会花费几年时间，但如果有成效则可能有助于非小细胞肺癌的早期诊断或更有效的治疗。

【替代治疗】

参加本研究是完全自愿的，如果您选择不参加、或在研究的任何阶段选择退出，不会影响您既定的治疗方案。

【新发现】

在研究期间，若有任何关于疾病或任何关于药物会影响您决定是否继续参加本研究的重要信息，您的医生将及时通知您。试验期间，您可随时了解与其有关的信息资料。

【请您配合的事项】

假使您选择参与本研究，研究期间请密切配合研究者的工作，并请遵守以下事项：

（1） 按时到医院随访；

（2） 请告诉医生试验过程的任何不适，以便医生对症处理；

（3） 您需要在研究过程中向医生或研究者提供有关您的健康资讯，特别是任何有利或不利的改变。

【保密】

您的病情信息在任何时候都会被保密。只有研究医生保留您的基本信息，在研究的其他文件中将使用您的姓名缩写和代码标识。除了您的研究医生外，医院的伦理委员会的代表可能将会查阅您与研究有关的原始医疗资料以确保研究是规范的，数据是真实可靠的。但所有的信息将会保密，本研究结果可能会发表在医学杂志上，但也不会泄露您的身份。

【参加原则】

您参加本研究完全是自愿的。您有权随时退出研究而您的医疗待遇与权益也不会受到影响。您有权了解有关的研究用药可能带来的不良反应。若继续参加研究会对您的健康造成伤害，或您的健康已不适合继续参加试验，或您不能遵守试验方案的要求，您的医生可以终止您的研究而不必得到您的同意。如果您决定退出研究，请您务必与负责试验的医生联系，医生将会安排您回诊一次以作各项指标的评估。

本研究的研究者也可能会出于安全或其它方面的考虑提前中止整个研究或对整个研究做出限制。

知情同意书签字页

我在充分了解受试者须知的全部内容以及参加本研究可能带来的利弊后，自愿参加本研究，并做出以下申明：

1. 作为受试者，我已阅读了上述受试者须知内容并理解本研究的性质、目的及该药物可能出现的不良反应等信息，我的问题已经得到满意的答复。
2. 我同意在研究中按时就诊随访，并接受与本研究有关的相应检查。我将遵守受试者须知要求，并与研究人员充分合作，如实、客观地向研究人员提供参加本研究前、研究期间和各随访期的健康状况及相关情况；
3. 我亦明白我可以随时退出研究，而此后的治疗并不会因此受到不利影响。我理解研究者有权根据我的情况随时终止研究；
4. 我明白我会收到一份签署过的知情同意书副本；
5. 我同意在此项医学研究中收集、使用和发表我的医学健康数据。
6. 经过充分考虑后，我自愿参加肺段切除术治疗 2-3cm 磨玻璃成分为主型浸润性肺癌的单臂、多中心、前瞻性、验证性 III 期临床试验。
7. 若我有任何关于研究和受试者权益的信息问题时，可以与以下研究人员联系：研究医生_傅方求_，电话_64175590-82500

受试者签名：____________________ **日期：**_____**年**____**月**____**日**

（仅当受试者不能阅读或签字时需法定监护人签名）

受试者法定监护人签名：______________ **日期：**_____**年**____**月**____**日**

我已对上述参加研究的自愿者解释了该项研究的有关细节，并且为他/她提供一份签署过的知情同意书副本。

研究者签名：______________ **日期：**_____**年**____**月**____**日**

Chen S, *et al. BMJ Open* 2024; 14:e087088. doi: 10.1136/bmjopen-2024-087088
