# Supplementary material for: Segmentectomy for ground glass-dominant invasive lung cancer with tumour diameter of 2–3 cm: protocol for a single-arm, multicentre, phase III trial (ECTOP1012)
Source: BMJ Open. 2024 Jul 3;14(7):e087088. doi: 10.1136/bmjopen-2024-087088 (PMC11227815; doi:10.1136/bmjopen-2024-087088)
Supplement: Author's manuscript [file bmjopen-2024-087088.draft_revisions.pdf]

BMJ Open is committed to open peer review. As part of this commitment we make the peer review history of every article we publish publicly available.

When an article is published we post the peer reviewers' comments and the authors' responses online. We also post the versions of the paper that were used during peer review. These are the versions that the peer review comments apply to.

The versions of the paper that follow are the versions that were submitted during the peer review process. They are not the versions of record or the final published versions. They should not be cited or distributed as the published version of this manuscript.

BMJ Open is an open access journal and the full, final, typeset and author-corrected version of record of the manuscript is available on our site with no access controls, subscription charges or pay-per-view fees (http://bmjopen.bmj.com).

If you have any questions on BMJ Open's open peer review process please email info.bmjopen@bmj.com

BMJ Open

Segmentectomy for ground glass-dominant invasive lung cancer with size of 2-3cm: protocol for a single-arm, multi-center, phase III trial (ECTOP1012)

Journal:	*BMJ Open*
Manuscript ID	bmjopen-2024-087088
Article Type:	Protocol
Date Submitted by the Author:	31-Mar-2024
Complete List of Authors:	Chen, Shiqi; Fudan University Shanghai Cancer Center, Department of Thoracic Surgery<br>Huang, Qingyuan; Fudan University Shanghai Cancer Center, Department of Thoracic Surgery<br>Fu, Fangqiu; Fudan University Shanghai Cancer Center, Department of Thoracic Surgery<br>Zhang, Yang; Fudan University Shanghai Cancer Center, Department of Thoracic Surgery<br>Chen, Haiquan ; Fudan University Shanghai Cancer Center, Thoracic Surgery
Keywords:	Clinical Trial, Lung Diseases, SURGERY

SCHOLARONE™
Manuscripts

Segmentectomy for ground glass-dominant invasive lung cancer with size of 2-3cm: protocol for a single-arm, multi-center, phase III trial (ECTOP1012)

Shiqi Chen[1,2,3#], Qingyuan Huang[1,2,3#], Fangqiu Fu[1,2,3#], Yang Zhang[1,2,3*], Haiquan Chen[1,2,3*]

Authors' Affiliations:

[1]Departments of Thoracic Surgery and State Key Laboratory of Genetic Engineering, Fudan University Shanghai Cancer Center, Shanghai, 200032, China;

[2]Institute of Thoracic Oncology, Fudan University, Shanghai, 200032, China;

[3]Department of Oncology, Shanghai Medical College, Fudan University, Shanghai, 200032, China.

Drs. Chen, Huang and Fu contributed equally to this work. Drs. Zhang and Chen contributed equally as co-senior and correspondence authors.

*Corresponding author: Haiquan Chen, E-mail: hqchen1@yahoo.com;

Yang Zhang, E-mail: fduzhangyang1987@hotmail.com

Abstract

Introduction: Previous studies demonstrated that wedge resection is sufficient for ground glass-dominant lung adenocarcinoma (LUAD) with tumor diameter ≤ 2cm, however, the optimal surgical type for ground glass-dominant LUAD with tumor diameter of 2-3cm remains unclear. The purpose of this trial is to investigate the safety and efficacy of segmentectomy for ground glass-dominant invasive LUAD with tumor size of 2-3cm.

Methods and analysis: In February 2023, recruitment began for this prospective, phase III trial to investigate whether segmentectomy is suitable for ground glass-dominant invasive LUAD with tumor size of 2-3cm. We plan to enroll a total of 307 patients from multiple institutions over a period of 5 years. The primary endpoint is 5-year disease-free survival (DFS); the secondary endpoints are postoperative pulmonary function, 5-year overall survival (OS), the site of tumor

For peer review only - http://bmjopen.bmj.com/site/about/guidelines.xhtml

recurrence and metastasis, segmentectomy completion rate, radical segmentectomy (R0 resection) completion rate, surgery-related complications. The statistician and the principal investigator shall formulate a statistical analysis scheme, and conduct statistical analysis after the trial is done.

Ethics and dissemination: This trial was approved by the institutional review board of each participating center. Patients need to sign written informed consent to participate in this trial. The research results will be published in a peer-reviewed international journal.

Trial registration number: NCT05717803.

Strengths and limitations of this study

- This is the first trial to investigate whether segmentectomy is sufficient for the specific group of patients of ground glass-dominant invasive LUAD with tumor size of 2-3cm.
- Only patients with invasive adenocarcinoma would be included, and the diagnosis is confirmed by intraoperative frozen sections and postoperative paraffin sections, thus restricting confounding pathological types.
- A limitation of the trial is its nonrandomized fashion. Considering the indolent nature and excellent prognosis of ground glass-dominant LUAD, lobectomy might be too invasive, so randomization would not be performed.

Word Count: 1643 words

Introduction

Lung cancer is the most commonly diagnosed cancer in men and the leading cause of cancer-associated mortality in both men and women[1]. Since the national lung screening trial (NLST) demonstrated that screening with low-dose computed tomography (LDCT) could increase the detection of lung cancer, and reduce the risk of mortality from lung cancer, LDCT is widely used in the screening for lung cancer[2]. LDCT screening leads to a dramatic increase in the detection of small-sized lung nodules, especially ground-glass opacity (GGO). The shift of spectrum of radiological characteristics challenges lobectomy as the “gold standard” of surgical treatment for

For peer review only

For peer review only - http://bmjopen.bmj.com/site/about/guidelines.xhtml

early-stage lung cancer, which was established in the 1990s by the Lung Cancer Study Group (LCSG) trial[3].

From 2002 to 2004, the JCOG0201 trial prospectively included patients with clinical T1N0M0 (≤3cm in tumor diameter) peripheral lung cancer, and defined radiological non-invasive lung adenocarcinoma (LUAD) as tumor size≤2cm with consolidation-to-tumor ratio (CTR) ≤0.25 [4]. Afterwards, the JCOG1211 trial found that the 5-year recurrence-free survival (RFS) of patients undergoing segmentectomy for the “radiologically invasive” tumors (tumor diameter≤2cm with CTR between 0.25 and 0.50, or tumor diameter between 2cm and 3cm with CTR ≤0.50) is 98.0%, exceeding the 87% of the pre-set threshold[5]. However, this trial was criticized that about half of included patients were pathologically confirmed as adenocarcinoma in situ (AIS) and microinvasive adenocarcinoma (MIA). For these patients (AIS/MIA), wedge resection is sufficient, and segmentectomy may result in overtreatment. Additionally, this also limits the statistical power to demonstrate the efficacy for pathologically invasive LUAD. Recently, our team performed a retrospective study and found that wedge resection for peripheral invasive LUAD with tumor diameter≤ 2 cm and CTR≤0.5 could lead to excellent long-term survival[6].

Therefore, a single-arm, multi-center, phase III trial (ECTOP1012) has been conducting to investigate whether segmentectomy is suitable for ground glass-dominant invasive lung cancer with tumor size of 2-3cm. We used the SPIRIT checklist when writing our report[7].

For peer review only

Methods and analysis

Study design

ECTOP1012 is a single-arm, multicenter, prospective, confirmatory phase III trial in order to evaluate the safety and efficacy of segmentectomy for ground glass-dominant invasive lung cancer with size of 2-3cm. As the prognosis of these patients is fairly excellent, a large sample size would be required if a randomized controlled trial (RCT) is to be performed, so this study is designed as a single-arm confirmatory trial. The patients' tumor size should be between 2 and 3 cm, and the CTR should not be greater than 0.5 on preoperative high-resolution CT (HRCT). The surgical type of lung tissue is segmentectomy, which includes single segment resection,

For peer review only - http://bmjopen.bmj.com/site/about/guidelines.xhtml

combined segment resection, tri-segmentectomy and basal segment resection. In addition, tumors located in the right middle lung are excluded in this trial (**Figure 1**).

Objectives

The primary objective of ECTOP1012 is to assess the efficacy of segmentectomy for ground glass-dominant invasive lung cancer with size of 2-3cm.

The secondary objectives include:

1. Surgical safety of segmentectomy for ground glass-dominant invasive lung cancer with size of 2-3cm.
2. Effect of segmentectomy on lung function.
3. Tumor recurrence rate after segmentectomy.

Endpoints

The primary endpoint is 5-year disease-free survival (5-year DFS). DFS events are defined as tumor recurrence due to lung cancer or death from any cause. Alive patients without recurrence at the last follow-up are defined as censored.

The secondary endpoints are postoperative pulmonary function, 5-year overall survival (5-year OS), the site of tumor recurrence and metastasis, segmentectomy completion rate, radical segmentectomy (R0 resection) completion rate, surgery-related complications.

Eligibility criteria

Inclusion criteria

All enrolled patients should meet the following criteria:

1) Voluntarily participate in this trial, willing to follow and capable to complete all trial procedures;

2) 18-80 years old (including cut-off value) at the time of signing the informed consent;

3) ECOG score of 0 or 1;

4) Have not undergone lung cancer surgery before;

5) Intraoperative or postoperative pathological confirmation of invasive lung adenocarcinoma;

6) A single pulmonary nodule with a predominantly ground-glass component or pure ground-glass on CT, or multiple pulmonary nodules but the main lesion is the above-mentioned nodule;

For peer review only - http://bmjopen.bmj.com/site/about/guidelines.xhtml

7) The consolidation-to-tumor ratio (CTR) of the nodule ranges from 0-0.5, and the total size of the nodule is between 2-3cm;

8) The preoperative lymph node stage is cN0 and there is no evidence of distant metastasis;

9) After the evaluation of the surgeon, the tumor can be completely removed by segmentectomy;

10) Treatment-naïve and no radiotherapy or chemotherapy.

Exclusion criteria

Any of the following are the exclusion criteria for this study:

1) CTR is not between 0-0.5 or the total nodule size is not between 2-3cm;

2) The lesion can't be completely surgically removed by segmentectomy;

3) Cytological or histopathological confirmation of other lung malignancies other than lung adenocarcinoma;

4) Previous lung cancer surgery;

5) Non-treatment-naïve patients who have received radiotherapy or chemotherapy.

Withdrawal or termination criteria

All data in this trial is obtained on the basis of existing examinations and procedures, and does not cause any additional harm to patients. The specifics of the patient's withdrawal from this study are:

1)The patient's data shall not be used for the clinical study.

2)Patient enrollment error (e.g., patient does not meet enrollment/exclusion criteria).

3)The patient voluntarily proposes to withdraw from the clinical study.

Treatment

Segmentectomy and lymph node dissection

The surgeon evaluates the lesions of the lung segment that can be completely resected would be included in the study. Segmentectomy with a smaller extent than lobectomy can be included in this study. Anatomical segmentectomy can be single segmentectomy (eg, S3 resection), combined segmentectomy (eg, S7+S8 resection), basal segmentectomy (eg, S7-S10 resection), and tri-segmentectomy (eg, left S1-S3 resection). In addition, the use of wedge resection to manage the patient's other non-major lesions is allowed in this study.

For peer review only - http://bmjopen.bmj.com/site/about/guidelines.xhtml

Based on the results of previous studies, there was no lymph node metastasis in lung cancer with a CTR <0.5[8], so there is no further provision for mediastinal lymph node dissection in this study, and the surgeon can perform it according to personal experience. The lymph nodes exposed during the operation need to be removed and sent for pathological examination.

Procedures

Preoperative CT imaging evaluation and clinical staging

All patients are required to undergo a thin-slice (1mm) contrast-enhanced CT scan of the chest before enrollment. According to the CT scan results, the long diameter of the tumor is the maximum diameter of the tumor (solid component + ground-glass component) on the CT cross-section. The size of the solid component is the maximum diameter of the solid component on the CT cross-section. Consolidation to tumor ratio (CTR) is defined as the ratio of solid component size to tumor diameter. For pure ground-glass nodules in which there are no solid component, the CTR is 0. cN0 is diagnosed when the short axis of lymph nodes of intrapulmonary, interlobard, hilar and mediastinal are shorter than 10 mm on contrast-enhanced CT, or no uptake of lymph nodes on PET/CT[9]. Lung nodules with a tumor diameter of 2-3cm, CTR≤0.5 and cN0 are included in the study. The staging in this study is performed using the 8th edition of the TNM lung cancer staging system.

Intraoperative frozen pathological evaluation criteria

All primary tumors are sent to the pathology room for frozen section diagnosis immediately after resection. Frozen pathology is taken from the maximum diameter of the tumor. Diagnosis is based on the 2015 WHO classification criteria. Intraoperative frozen lesions diagnosed as invasive lung adenocarcinoma/non-small cell lung cancer are included in this study.

Postoperative pathological evaluation criteria

The postoperative pathological diagnosis of tumor using paraffin sections is based on the 2015 version of the WHO classification criteria, and patients with lung adenocarcinoma are included in this study. All pathology reports are evaluated by two experienced pathologists.

Statistical analysis

Statistical plan

For peer review only - http://bmjopen.bmj.com/site/about/guidelines.xhtml

The statistician and the principal investigator shall formulate a statistical analysis plan according to the research plan, and conduct statistical analysis according to the statistical plan after the trial is done.

Data selection for statistical analysis

Full Analysis Set (FAS): It is a dataset determined according to the ITT principle, including all enrolled cases who have undergone segmentectomy.

Per-Protocol Set (PPS): It is a subset of the FAS population, including all cases that meet the protocol and have completed CRF.

In this trial, FAS analysis is used for baseline data and validity analysis. The main efficacy indicators are also analyzed on the PPS dataset. However, the conclusions of the FAS dataset are the main ones. When FAS and PPS reach consistent conclusions, confidence in the conclusions can be increased. For the FAS set, the missing data after the fall is estimated using the LOCF method. The laboratory examination data and adverse events are analyzed by SPSS and STATA software.

Calculation of sample size

According to the results of previous studies, for patients undergoing lobectomy with tumors size of 2-3 cm, and CTR$\leqslant$0.5, and the 5-year DFS was 97.0% after surgery. We estimate that the expected 5-year DFS for these patients undergoing segmentectomy is 95%, with the lower limit defined as 90%. Taking 5% as the non-inferior boundary, a total of 307 cases needs to be included in this trial according to the dropout rate of 5%.

Ethics and dissemination

Only patients who can fully understand the risks, benefits, and potential adverse events of this trial will be enrolled after voluntarily signing an informed consent form. The protocol and informed consent form had been reviewed and approved by the institutional review board of each participating center (such as: the Ethics Committee of Fudan University Shanghai Cancer Center). Informed consent should be in line with the Declaration of Helsinki. The personal data collected and processed by the patients enrolled in this study will be limited to those necessary to complete the purposes of this study. The clinical trial was registered on February 8 2023 on

For peer review only

For peer review only - http://bmjopen.bmj.com/site/about/guidelines.xhtml

ClinicalTrials.gov (NCT05717803). The results of the trial will be added on ClinicalTrials.gov, published in peer-reviewed journals and presented at international conferences.

Author Contributions

HC and YZ: conceiving the study. SC, QH and FF: drafting the protocol. QH and FF: registering the protocol and ethical submission. QH and HC: funding acquisition. YZ and HC: supervision and revising the draft. All authors contributed to the article and approved the submitted version.

Funding statement

This work was supported by the National Natural Science Foundation of China (81930073, 82203504), the Shanghai Technology Innovation Action Project (20JC1417200), the Cooperation Project of Conquering Major Diseases in Xuhui District (XHLHGG202101), and the National Key R&D Program of China (2022YFA1103900).

Competing interests statement: None declared.

Data access: The Data Management Coordinating Center will oversee the intra-study data sharing process, with input from the Data Management Subcommittee. All Principal Investigators will be given access to the cleaned data sets. Project data sets will be housed on the Project Accept Web site and/or the file transfer protocol site created for the study, and all data sets will be password protected. Project Principal Investigators will have direct access to their own site's data sets, and will have access to other sites data by request. To ensure confidentiality, data dispersed to project team members will be blinded of any identifying participant information.

For peer review only

For peer review only - http://bmjopen.bmj.com/site/about/guidelines.xhtml

References:

[1] SIEGEL R L, MILLER K D, FUCHS H E, et al. Cancer statistics, 2022 [J]. CA Cancer J Clin, 2022, 72(1): 7-33.

[2] ABERLE D R, BERG C D, BLACK W C, et al. The National Lung Screening Trial: overview and study design [J]. Radiology, 2011, 258(1): 243-53.

[3] GINSBERG R J, RUBINSTEIN L. The comparison of limited resection to lobectomy for T1N0 non-small cell lung cancer. LCSG 821 [J]. Chest, 1994, 106(6 Suppl): 318s-9s.

[4] SUZUKI K, KOIKE T, ASAKAWA T, et al. A prospective radiological study of thin-section computed tomography to predict pathological noninvasiveness in peripheral clinical IA lung cancer (Japan Clinical Oncology Group 0201) [J]. Journal of thoracic oncology : official publication of the International Association for the Study of Lung Cancer, 2011, 6(4): 751-6.

[5] AOKAGE K, SAJI H, SUZUKI K, et al. A non-randomized confirmatory trial of segmentectomy for clinical T1N0 lung cancer with dominant ground glass opacity based on thin-section computed tomography (JCOG1211) [J]. General thoracic and cardiovascular surgery, 2017, 65(5): 267-72.

[6] ZHANG C, PAN Y, LI H, et al. Extent of surgical resection for radiologically subsolid T1N0 invasive lung adenocarcinoma: When is a wedge resection acceptable? [J]. The Journal of thoracic and cardiovascular surgery, 2024, 167(3): 797-809.e2.

[7] CHAN A W, TETZLAFF J M, GøTZSCHE P C, et al. SPIRIT 2013 explanation and elaboration: guidance for protocols of clinical trials [J]. Bmj, 2013, 346: e7586.

[8] ZHANG Y, FU F, WEN Z, et al. Segment Location and Ground Glass Opacity Ratio Reliably Predict Node-Negative Status in Lung Cancer [J]. The Annals of thoracic surgery, 2020, 109(4): 1061-8.

[9] DARLING G E, MAZIAK D E, INCULET R I, et al. Positron emission tomography-computed tomography compared with invasive mediastinal staging in non-small cell lung cancer: results of mediastinal staging in the early lung positron emission tomography trial [J]. Journal of thoracic oncology : official publication of the International Association for the Study of Lung Cancer, 2011, 6(8): 1367-72.

For peer review only - http://bmjopen.bmj.com/site/about/guidelines.xhtml

Figure1. Flowchart of ECTOP1012.

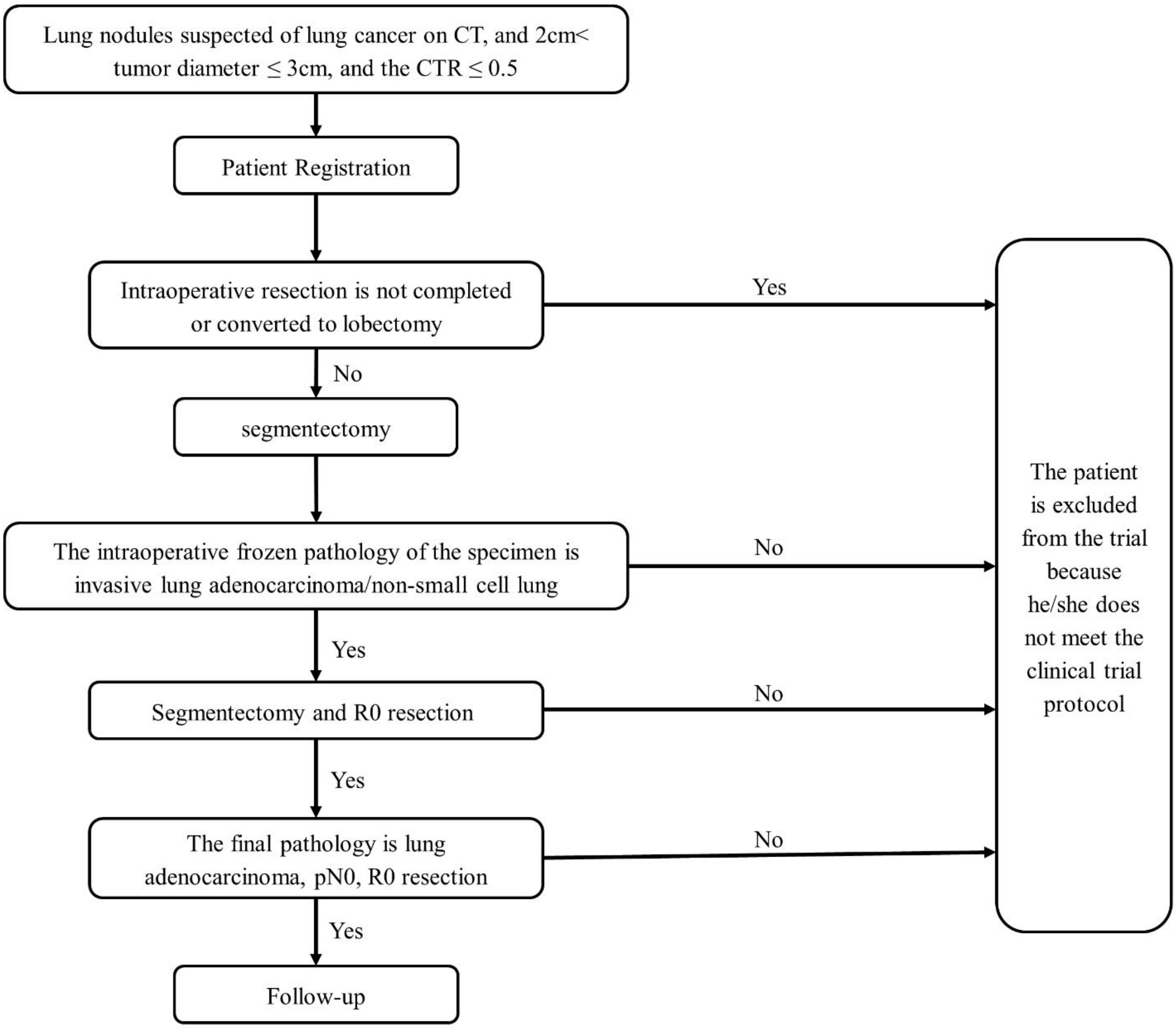

Reporting checklist for protocol of a clinical trial.

Based on the SPIRIT guidelines.

Instructions to authors

Complete this checklist by entering the page numbers from your manuscript where readers will find each of the items listed below.

Your article may not currently address all the items on the checklist. Please modify your text to include the missing information. If you are certain that an item does not apply, please write "n/a" and provide a short explanation.

Upload your completed checklist as an extra file when you submit to a journal.

In your methods section, say that you used the SPIRITreporting guidelines, and cite them as:

Chan A-W, Tetzlaff JM, Gøtzsche PC, Altman DG, Mann H, Berlin J, Dickersin K, Hróbjartsson A, Schulz KF, Parulekar WR, Krleža-Jerić K, Laupacis A, Moher D. SPIRIT 2013 Explanation and Elaboration: Guidance for protocols of clinical trials. BMJ. 2013;346:e7586

		Reporting Item	Page Number
**Administrative information**			
Title	#1	Descriptive title identifying the study design, population, interventions, and, if applicable, trial acronym	1-2
Trial registration	#2a	Trial identifier and registry name. If not yet registered, name of intended registry	2
Trial registration: data set	#2b	All items from the World Health Organization Trial Registration Data Set	n/a
Protocol version	#3	Date and version identifier	n/a
Funding	#4	Sources and types of financial, material, and other support	8
Roles and responsibilities: contributorship	#5a	Names, affiliations, and roles of protocol contributors	8

For peer review only

Roles and responsibilities: sponsor contact information	#5b	Name and contact information for the trial sponsor	1
Roles and responsibilities: sponsor and funder	#5c	Role of study sponsor and funders, if any, in study design; collection, management, analysis, and interpretation of data; writing of the report; and the decision to submit the report for publication, including whether they will have ultimate authority over any of these activities	8
Roles and responsibilities: committees	#5d	Composition, roles, and responsibilities of the coordinating centre, steering committee, endpoint adjudication committee, data management team, and other individuals or groups overseeing the trial, if applicable (see Item 21a for data monitoring committee)	n/a
**Introduction**			
Background and rationale	#6a	Description of research question and justification for undertaking the trial, including summary of relevant studies (published and unpublished) examining benefits and harms for each intervention	2-3
Background and rationale: choice of comparators	#6b	Explanation for choice of comparators	2-3
Objectives	#7	Specific objectives or hypotheses	2-3
Trial design	#8	Description of trial design including type of trial (eg, parallel group, crossover, factorial, single group), allocation ratio, and framework (eg, superiority, equivalence, non-inferiority, exploratory)	2-3
**Methods: Participants, interventions, and outcomes**			
Study setting	#9	Description of study settings (eg, community clinic, academic hospital) and list of countries where data will be collected. Reference to where list of study sites can be obtained	3-4
Eligibility criteria	#10	Inclusion and exclusion criteria for participants. If applicable, eligibility criteria for study centres and individuals who will	4-5

For peer review only

		perform the interventions (eg, surgeons, psychotherapists)	
Interventions: description	#11a	Interventions for each group with sufficient detail to allow replication, including how and when they will be administered	6
Interventions: modifications	#11b	Criteria for discontinuing or modifying allocated interventions for a given trial participant (eg, drug dose change in response to harms, participant request, or improving / worsening disease)	6
Interventions: adherance	#11c	Strategies to improve adherence to intervention protocols, and any procedures for monitoring adherence (eg, drug tablet return; laboratory tests)	6
Interventions: concomitant care	#11d	Relevant concomitant care and interventions that are permitted or prohibited during the trial	6
Outcomes	#12	Primary, secondary, and other outcomes, including the specific measurement variable (eg, systolic blood pressure), analysis metric (eg, change from baseline, final value, time to event), method of aggregation (eg, median, proportion), and time point for each outcome. Explanation of the clinical relevance of chosen efficacy and harm outcomes is strongly recommended	4
Participant timeline	#13	Time schedule of enrolment, interventions (including any run-ins and washouts), assessments, and visits for participants. A schematic diagram is highly recommended (see Figure)	4
Sample size	#14	Estimated number of participants needed to achieve study objectives and how it was determined, including clinical and statistical assumptions supporting any sample size calculations	7
Recruitment	#15	Strategies for achieving adequate participant enrolment to reach target sample size	7
**Methods: Assignment of interventions (for controlled trials)**			
Allocation: sequence generation	#16a	Method of generating the allocation sequence (eg, computer-generated random numbers), and list of any factors for stratification. To reduce predictability of a random sequence, details of any planned restriction (eg, blocking) should be provided in a separate document that is unavailable to those who enrol participants or assign interventions	n/a

For peer review only

Allocation concealment mechanism	#16b	Mechanism of implementing the allocation sequence (eg, central telephone; sequentially numbered, opaque, sealed envelopes), describing any steps to conceal the sequence until interventions are assigned	n/a
Allocation: implementation	#16c	Who will generate the allocation sequence, who will enrol participants, and who will assign participants to interventions	n/a
Blinding (masking)	#17a	Who will be blinded after assignment to interventions (eg, trial participants, care providers, outcome assessors, data analysts), and how	n/a
Blinding (masking): emergency unblinding	#17b	If blinded, circumstances under which unblinding is permissible, and procedure for revealing a participant's allocated intervention during the trial	n/a
**Methods: Data collection, management, and analysis**			
Data collection plan	#18a	Plans for assessment and collection of outcome, baseline, and other trial data, including any related processes to promote data quality (eg, duplicate measurements, training of assessors) and a description of study instruments (eg, questionnaires, laboratory tests) along with their reliability and validity, if known. Reference to where data collection forms can be found, if not in the protocol	7
Data collection plan: retention	#18b	Plans to promote participant retention and complete follow-up, including list of any outcome data to be collected for participants who discontinue or deviate from intervention protocols	7
Data management	#19	Plans for data entry, coding, security, and storage, including any related processes to promote data quality (eg, double data entry; range checks for data values). Reference to where details of data management procedures can be found, if not in the protocol	7
Statistics: outcomes	#20a	Statistical methods for analysing primary and secondary outcomes. Reference to where other details of the statistical analysis plan can be found, if not in the protocol	7
Statistics: additional analyses	#20b	Methods for any additional analyses (eg, subgroup and adjusted analyses)	7

For peer review only

Statistics: analysis population and missing data	#20c	Definition of analysis population relating to protocol non-adherence (eg, as randomised analysis), and any statistical methods to handle missing data (eg, multiple imputation)	7
**Methods: Monitoring**			
Data monitoring: formal committee	#21a	Composition of data monitoring committee (DMC); summary of its role and reporting structure; statement of whether it is independent from the sponsor and competing interests; and reference to where further details about its charter can be found, if not in the protocol. Alternatively, an explanation of why a DMC is not needed	7
Data monitoring: interim analysis	#21b	Description of any interim analyses and stopping guidelines, including who will have access to these interim results and make the final decision to terminate the trial	7
Harms	#22	Plans for collecting, assessing, reporting, and managing solicited and spontaneously reported adverse events and other unintended effects of trial interventions or trial conduct	n/a
Auditing	#23	Frequency and procedures for auditing trial conduct, if any, and whether the process will be independent from investigators and the sponsor	n/a
**Ethics and dissemination**			
Research ethics approval	#24	Plans for seeking research ethics committee / institutional review board (REC / IRB) approval	7
Protocol amendments	#25	Plans for communicating important protocol modifications (eg, changes to eligibility criteria, outcomes, analyses) to relevant parties (eg, investigators, REC / IRBs, trial participants, trial registries, journals, regulators)	n/a
Consent or assent	#26a	Who will obtain informed consent or assent from potential trial participants or authorised surrogates, and how (see Item 32)	7
Consent or assent: ancillary studies	#26b	Additional consent provisions for collection and use of participant data and biological specimens in ancillary studies, if applicable	n/a
Confidentiality	#27	How personal information about potential and enrolled participants will be collected, shared, and maintained in order to protect confidentiality before, during, and after the trial	7

For peer review only

Declaration of interests	#28	Financial and other competing interests for principal investigators for the overall trial and each study site	8
Data access	#29	Statement of who will have access to the final trial dataset, and disclosure of contractual agreements that limit such access for investigators	8
Ancillary and post trial care	#30	Provisions, if any, for ancillary and post-trial care, and for compensation to those who suffer harm from trial participation	n/a
Dissemination policy: trial results	#31a	Plans for investigators and sponsor to communicate trial results to participants, healthcare professionals, the public, and other relevant groups (eg, via publication, reporting in results databases, or other data sharing arrangements), including any publication restrictions	7-8
Dissemination policy: authorship	#31b	Authorship eligibility guidelines and any intended use of professional writers	n/a
Dissemination policy: reproducible research	#31c	Plans, if any, for granting public access to the full protocol, participant-level dataset, and statistical code	n/a
**Appendices**			
Informed consent materials	#32	Model consent form and other related documentation given to participants and authorised surrogates	n/a
Biological specimens	#33	Plans for collection, laboratory evaluation, and storage of biological specimens for genetic or molecular analysis in the current trial and for future use in ancillary studies, if applicable	n/a

The SPIRIT Explanation and Elaboration paper is distributed under the terms of the Creative Commons Attribution License CC-BY-NC. This checklist was completed on 30. March 2024 using https://www.goodreports.org/, a tool made by the EQUATOR Network in collaboration with Penelope.ai

For peer review only

BMJ Open

Segmentectomy for ground glass-dominant invasive lung cancer with tumour diameter of 2-3 cm: protocol for a single-arm, multicentre, phase III trial (ECTOP1012)

Journal:	*BMJ Open*
Manuscript ID	bmjopen-2024-087088.R1
Article Type:	Protocol
Date Submitted by the Author:	06-Jun-2024

Complete List of Authors:	Chen, Shiqi; Fudan University Shanghai Cancer Center, Department of Thoracic Surgery<br>Huang, Qingyuan; Fudan University Shanghai Cancer Center, Department of Thoracic Surgery<br>Fu, Fangqiu; Fudan University Shanghai Cancer Center, Department of Thoracic Surgery<br>Wang, Zezhou; Fudan University Shanghai Cancer Center, Department of Cancer Prevention<br>Zhang, Yang; Fudan University Shanghai Cancer Center, Department of Thoracic Surgery<br>Chen, Haiquan ; Fudan University Shanghai Cancer Center, Thoracic Surgery

**Primary Subject Heading**:	Surgery
Secondary Subject Heading:	Oncology
Keywords:	Clinical Trial, Lung Diseases, SURGERY

SCHOLARONE™
Manuscripts

Segmentectomy for ground glass-dominant invasive lung cancer with tumour diameter of 2-3 cm: protocol for a single-arm, multicentre, phase III trial (ECTOP1012)

Shiqi Chen[1,2,3#], Qingyuan Huang[1,2,3#], Fangqiu Fu[1,2,3#], Zezhou Wang[3,4], Yang Zhang[1,2,3*], Haiquan Chen[1,2,3*]

Authors' affiliations:

[1]Departments of Thoracic Surgery and State Key Laboratory of Genetic Engineering, Fudan University Shanghai Cancer Center, Shanghai, 200032, China;

[2]Institute of Thoracic Oncology, Fudan University, Shanghai, 200032, China;

[3]Department of Oncology, Shanghai Medical College, Fudan University, Shanghai, 200032, China.

[4]Department of Cancer Prevention, Fudan University Shanghai Cancer Center, Shanghai, 200032, China.

#Drs. Chen, Huang, and Fu contributed equally to this work.

*Drs. Zhang and Chen contributed equally as co-senior and corresponding authors.

Correspondence to:

Haiquan Chen, E-mail: hqchen1@yahoo.com

Yang Zhang, E-mail: fduzhangyang1987@hotmail.com

Abstract

Introduction: Previous studies demonstrated that wedge resection is sufficient for ground glass-dominant lung adenocarcinoma (LUAD) with tumour diameter ≤ 2 cm, however, the optimal surgical type for ground glass-dominant LUAD with tumour diameter of 2-3 cm remains unclear.

For peer review only - http://bmjopen.bmj.com/site/about/guidelines.xhtml

The purpose of this trial is to investigate the safety and efficacy of segmentectomy for ground glass-dominant invasive LUAD with tumour size of 2-3 cm.

Methods and analysis: We initiated a phase III trial to investigate whether segmentectomy is suitable for ground glass-dominant invasive LUAD with tumour size of 2-3 cm. This trial plans to enrol 307 patients from multiple institutions including four general hospitals and two specialty cancer hospitals over a period of 5 years. The primary endpoint is 5-year disease-free survival (DFS). Secondary endpoints are lung function, 5-year overall survival (OS), the site of tumour recurrence and metastasis, segmentectomy completion rate, radical segmentectomy (R0 resection) completion rate, and surgery-related complications.

Ethics and dissemination: This trial has been approved by the Ethics Committee of Fudan University Shanghai Cancer Center (reference 2212267-18) and by the institutional review boards of each participating centre. Written informed consent is required from all participants. The study results will be published in a peer-reviewed international journal.

Trial registration: ClinicalTrials.gov, NCT05717803.

Strengths and limitations of this study

- The trial is conducted in multiple centres, increasing the generalisability of the results.
- Both intraoperative frozen sections and postoperative paraffin sections will be evaluated by two experienced pathologists, making the pathologic diagnosis reliable.
- A key limitation of the trial is lack of a comparator group; considering the indolent nature and excellent prognosis of ground glass-dominant lung adenocarcinoma, lobectomy might be too invasive, so a control-arm of lobectomy is not set.

INTRODUCTION

Lung cancer is the most commonly diagnosed malignancy in men and the leading cause of cancer-associated mortality in both men and women(1). Since the national lung screening trial (NLST) demonstrated that screening with low-dose computed tomography (LDCT) could increase the detection of lung cancer, and reduce the risk of mortality from lung cancer, LDCT is

For peer review only - http://bmjopen.bmj.com/site/about/guidelines.xhtml

widely used in the screening for lung cancer(2). LDCT screening leads to a dramatic increase in the detection of small-sized lung nodules, especially ground-glass opacity (GGO)(3). The shift of the spectrum of radiological characteristics challenges lobectomy as the "gold standard" of surgical treatment for early-stage lung cancer, which was established in the 1990s by the Lung Cancer Study Group (LCSG) trial(4).

From 2002 to 2004, the JCOG0201 trial included patients with clinical T1N0M0 (≤3 cm in tumour diameter) peripheral lung cancer, and defined radiological non-invasive lung adenocarcinoma (LUAD) as tumour size ≤2 cm with consolidation-to-tumour ratio (CTR) ≤0.25 (5). Afterward, the JCOG1211 trial found that the 5-year recurrence-free survival (RFS) of patients undergoing segmentectomy for the "radiologically invasive" tumours (tumour diameter ≤2 cm with CTR between 0.25 and 0.50, or tumour diameter between 2 cm and 3 cm with CTR ≤0.50) is 98.0%, exceeding the 87% of the pre-set threshold(6). However, this trial was criticised on the basis that about half of the included patients were pathologically confirmed as adenocarcinoma in situ (AIS) and microinvasive adenocarcinoma (MIA). For these patients (AIS/MIA), wedge resection is sufficient, and segmentectomy may be unnecessary. Additionally, this also limits the statistical power to demonstrate the efficacy of pathologically invasive LUAD. Recently, our team performed a retrospective study and found that wedge resection for peripheral invasive LUAD with tumour diameter ≤ 2 cm and CTR≤0.5 could lead to excellent long-term survival(7).

This single-arm, multicentre, phase III trial (ECTOP1012) aims to investigate whether segmentectomy is suitable for ground glass-dominant invasive lung cancer with tumour size of 2-3 cm.

METHODS AND ANALYSIS

Study design

ECTOP1012 is a single-arm, multicentre, confirmatory phase III trial to evaluate the safety and efficacy of segmentectomy for ground glass-dominant invasive lung cancer with size of 2-3 cm. Four general hospitals and two specialty cancer hospitals will participate in this trial. As the prognosis of these patients is fairly excellent, a large sample size would be required if a

For peer review only - http://bmjopen.bmj.com/site/about/guidelines.xhtml

randomised controlled trial (RCT) is to be performed, so this study is designed as a single-arm confirmatory trial. The tumour size should be between 2 and 3 cm, and the CTR should not be greater than 0.5 on preoperative high-resolution CT (HRCT). The surgical procedure for included patients is segmentectomy, which includes single-segment resection, combined segment resection, tri-segmentectomy, and basal segment resection. Tumours located in the right middle lobe are excluded in this trial (**Figure 1**).

The trial started in February 2023; the estimated primary completion date is December 2028 and the estimated study completion date is December 2029. We used the SPIRIT checklist when preparing this report(8).

Objectives

The primary objective of ECTOP1012 is to assess the efficacy of segmentectomy for ground glass-dominant invasive lung cancer with size of 2-3 cm.

The secondary objectives include:

1. Surgical safety of segmentectomy for ground glass-dominant invasive lung cancer with size of 2-3 cm.
2. Loss of lung function after segmentectomy.
3. Tumour recurrence rate after segmentectomy.

Endpoints

The primary endpoint is 5-year disease-free survival (5-year DFS). DFS events are defined as tumour recurrence due to lung cancer or death from any cause. Alive patients without recurrence at the last follow-up are defined as censored.

The secondary endpoints are lung function, 5-year overall survival (5-year OS), the site of tumour recurrence and metastasis, segmentectomy completion rate, radical segmentectomy (R0 resection) completion rate, and surgery-related complications.

Eligibility criteria

Inclusion criteria

All enrolled patients should meet the following criteria:

1) Written informed consent;

For peer review only - http://bmjopen.bmj.com/site/about/guidelines.xhtml

114 2) 18-80 years old (including cut-off value) at the time of signing the informed consent;

115 3) ECOG score of 0 or 1;

116 4) No prior lung cancer surgery;

117 5) Invasive LUAD confirmed by intraoperative or postoperative pathological evaluation;

118 6) A single pulmonary nodule with a predominantly ground-glass component or pure ground-glass on CT, or multiple pulmonary nodules but the main lesion is the above-mentioned nodule;

120 7) The consolidation-to-tumour ratio (CTR) of the nodule ranges from 0-0.5, and the total size is between 2-3 cm;

122 8) No evidence of nodal or distant metastasis;

123 9) The tumour is evaluated to be completely removed via segmentectomy by thoracic surgeon;

124 10) No prior radiotherapy or chemotherapy.

125 ***Exclusion criteria***

126 Patients are excluded if they meet any of the following criteria:1) CTR is not between 0-0.5 or the total nodule size is not between 2-3 cm;

128 2) The lesion can't be completely surgically removed by segmentectomy;

129 3) Lung malignancies other than lung adenocarcinoma confirmed by cytological or histopathological evaluation;

131 4) Prior lung cancer surgery;

132 5) Non-treatment-naïve patients.

133 **Withdrawal or termination criteria**

134 All data in this trial is obtained based on existing examinations and procedures and the trial does not cause any additional harm to patients. The specifics of the patient's withdrawal from this study are:

137 1) The patient's data shall not be used for the clinical study.

138 2) Patient enrolment error (e.g., patient does not meet enrolment criteria).

139 3) Patients voluntarily propose to withdraw from the trial.

141 **Treatment**

142 ***Segmentectomy and lymph node dissection***

For peer review only - http://bmjopen.bmj.com/site/about/guidelines.xhtml

Pulmonary GGOs that are evaluated to be removed via segmentectomy by thoracic surgeons could be included in the study. Anatomical segmentectomy could be single segmentectomy (eg, S3 resection), combined segmentectomy (eg, S7+S8 resection), basal segmentectomy (eg, S7-S10 resection), and tri-segmentectomy (eg, left S1-S3 resection). In addition, the use of wedge resection to manage the patient's other non-major lesions is allowed in this study.

Based on the results of previous studies, there was no lymph node metastasis in lung cancer with a CTR <0.5(9), so there is no further provision for mediastinal lymph node dissection in this study, and the surgeon could choose the extent of mediastinal lymph node dissection according to personal experience. The lymph nodes exposed during the operation need to be removed and sent for pathological examination.

Procedures

Preoperative CT imaging evaluation and clinical staging

All patients are required to undergo a thin-slice (1mm) contrast-enhanced CT scan of the chest before enrolment. According to the CT scan results, the long diameter of the tumour is the maximum diameter of the tumour (solid component + ground-glass component) on the CT cross-section. The size of the solid component is the maximum diameter of the solid component on the CT cross-section. Consolidation to tumour ratio (CTR) is defined as the ratio of solid component size to tumour diameter. For pure ground-glass nodules in which there are no solid components, the CTR is 0. cN0 is diagnosed when the short axis of lymph nodes of intrapulmonary, interlobar, hilar, and mediastinal are shorter than 10 mm on contrast-enhanced CT, or no uptake of lymph nodes on PET/CT(10). The staging in this study is performed using the 8th edition of the TNM lung cancer staging system(11). Patients will be admitted to the hospital for surgery after the CT or PET-CT is evaluated by the radiologist, and the interval is one month.

Intraoperative frozen pathological evaluation criteria

All primary tumours are sent to the pathology room for frozen section diagnosis immediately after resection. Frozen pathology is taken from the maximum diameter of the tumour. Diagnosis is based on the 2015 WHO classification criteria(12). Lesions diagnosed as invasive lung adenocarcinoma/non-small cell lung cancer by intraoperative frozen are included in this study.

For peer review only - http://bmjopen.bmj.com/site/about/guidelines.xhtml

Postoperative pathological evaluation criteria

The postoperative pathological diagnosis of the tumour using paraffin sections is based on the 2015 version of the WHO classification criteria, and patients with lung adenocarcinoma are included in this study. All pathology reports are evaluated by two experienced pathologists.

Statistical analysis

Analysis sets

Full Analysis Set (FAS): Determined according to the ITT principle, including all enrolled cases who have undergone segmentectomy.

Per-Protocol Set (PPS): Subset of the FAS population, including all cases that meet the protocol and have completed CRF.

In this trial, FAS analysis is used for baseline data and validity analysis. The main efficacy indicators are will be analysed in the FAS and PPS datasets. However, the analyses of the FAS dataset are considered primary. When analyses of the FAS and PPS reach consistent conclusions, confidence in the conclusions can be increased. For the FAS dataset, the missing data after the fall is estimated using the LOCF method.

The laboratory examination data and adverse events will be analysed using SPSS and Stata software.

Calculation of sample size

According to the results of previous studies, for patients undergoing lobectomy with tumour sizes of 2-3 cm, CTR≤0.5, and the 5-year DFS was 97.0% after surgery(13). In this trial, we assumed that the expected 5-year DFS for these patients undergoing segmentectomy is 95%. A sample size of 292 achieves 90.193% power to detect a non-inferiority proportion of 90% (5% as the non-inferior boundary) using a one-sided exact test with a target significance level of 0.0250. Considering a possible dropout rate of 5%, a total of 307 cases need to be included in this trial.

Data access

The Data Management Coordinating Center will oversee the intra-study data- sharing process, with input from the Data Management Subcommittee. All Principal Investigators will be given

For peer review only

For peer review only - http://bmjopen.bmj.com/site/about/guidelines.xhtml

access to the cleaned data sets. Project data sets will be housed on the Project Accept website and/or the file transfer protocol site created for the study, and all data sets will be password-protected. Project Principal Investigators will have direct access to their own site's data sets and will have access to other sites' data by request. To ensure confidentiality, data dispersed to project team members will be blinded to any identifying participant information.

Patient and public involvement

None.

ETHICS AND DISSEMINATION

The protocol and informed consent form have been reviewed and approved by the Ethics Committee of Fudan University Shanghai Cancer Center (reference 2212267-18) and by the institutional review boards of each participating centre. Only patients who can fully understand the risks, benefits, and potential adverse events of this trial will be enrolled after voluntarily signing an informed consent form (Supplemental Material 1). Informed consent should be in line with the Declaration of Helsinki.

The personal data collected and processed by the patients enrolled in this study will be limited to those necessary to complete the purposes of this study.

The trial was registered at ClinicalTrials.gov on February 8, 2023 (NCT05717803).

The results of the trial will be updated on ClinicalTrials.gov, published in peer-reviewed journals, and presented at international academic conferences.

Contributors

HC and YZ: conceiving the study. SC, QH, and FF: drafting the protocol. QH and FF: registering the protocol and ethical submission. QH and HC: funding acquisition. YZ and HC: supervision and revising the draft. ZW: calculation of sample size and statistical analysis interpretation. All authors contributed to the article and approved the submitted version. HC is the guarantor.

Funding

For peer review only - http://bmjopen.bmj.com/site/about/guidelines.xhtml

This work was supported by the National Natural Science Foundation of China (81930073, 82203504), the Shanghai Technology Innovation Action Project (20JC1417200), the Cooperation Project of Conquering Major Diseases in Xuhui District (XHLHGG202101), and the National Key R&D Program of China (2022YFA1103900).

Competing interests

None declared.

References

1. Siegel RL, Miller KD, Fuchs HE, Jemal A. Cancer statistics, 2022. CA Cancer J Clin. 2022;72(1):7-33.
2. Aberle DR, Berg CD, Black WC, Church TR, Fagerstrom RM, Galen B, et al. The National Lung Screening Trial: overview and study design. Radiology. 2011;258(1):243-53.
3. Li N, Tan F, Chen W, Dai M, Wang F, Shen S, et al. One-off low-dose CT for lung cancer screening in China: a multicentre, population-based, prospective cohort study. The Lancet Respiratory medicine. 2022;10(4):378-91.
4. Ginsberg RJ, Rubinstein L. The comparison of limited resection to lobectomy for T1N0 non-small cell lung cancer. LCSG 821. Chest. 1994;106(6 Suppl):318s-9s.
5. Suzuki K, Koike T, Asakawa T, Kusumoto M, Asamura H, Nagai K, et al. A prospective radiological study of thin-section computed tomography to predict pathological noninvasiveness in peripheral clinical IA lung cancer (Japan Clinical Oncology Group 0201). Journal of thoracic oncology : official publication of the International Association for the Study of Lung Cancer. 2011;6(4):751-6.
6. Aokage K, Suzuki K, Saji H, Wakabayashi M, Kataoka T, Sekino Y, et al. Segmentectomy for ground-glass-dominant lung cancer with a tumour diameter of 3 cm or less including ground-glass opacity (JCOG1211): a multicentre, single-arm, confirmatory, phase 3 trial. The Lancet Respiratory medicine. 2023;11(6):540-9.
7. Zhang C, Pan Y, Li H, Zhang Y, Li B, Zhang Y, et al. Extent of surgical resection for radiologically subsolid T1N0 invasive lung adenocarcinoma: When is a wedge resection acceptable? The Journal of thoracic and cardiovascular surgery. 2024;167(3):797-809.e2.
8. Chan AW, Tetzlaff JM, Gøtzsche PC, Altman DG, Mann H, Berlin JA, et al. SPIRIT 2013 explanation and elaboration: guidance for protocols of clinical trials. Bmj. 2013;346:e7586.
9. Zhang Y, Fu F, Wen Z, Deng L, Wang S, Li Y, et al. Segment Location and Ground Glass Opacity Ratio Reliably Predict Node-Negative Status in Lung Cancer. The Annals of thoracic surgery. 2020;109(4):1061-8.
10. Darling GE, Maziak DE, Inculet RI, Gulenchyn KY, Driedger AA, Ung YC, et al. Positron emission tomography-computed tomography compared with invasive mediastinal staging in non-small cell lung cancer: results of mediastinal staging in the early lung positron emission tomography trial. Journal of thoracic oncology : official publication of the International Association for the Study of Lung Cancer. 2011;6(8):1367-72.
11. Goldstraw P, Chansky K, Crowley J, Rami-Porta R, Asamura H, Eberhardt WE, et al. The IASLC Lung Cancer Staging Project: Proposals for Revision of the TNM Stage Groupings in the Forthcoming

For peer review only - http://bmjopen.bmj.com/site/about/guidelines.xhtml

(Eighth) Edition of the TNM Classification for Lung Cancer. Journal of thoracic oncology : official publication of the International Association for the Study of Lung Cancer. 2016;11(1):39-51.

12. Travis WD, Brambilla E, Nicholson AG, Yatabe Y, Austin JHM, Beasley MB, et al. The 2015 World Health Organization Classification of Lung Tumors: Impact of Genetic, Clinical and Radiologic Advances Since the 2004 Classification. Journal of thoracic oncology : official publication of the International Association for the Study of Lung Cancer. 2015;10(9):1243-60.

13. Ye T, Deng L, Wang S, Xiang J, Zhang Y, Hu H, et al. Lung Adenocarcinomas Manifesting as Radiological Part-Solid Nodules Define a Special Clinical Subtype. Journal of thoracic oncology: official publication of the International Association for the Study of Lung Cancer. 2019;14(4):617-27.

FIGURE TITLE

Figure 1. Study flowchart

For peer review only

For peer review only - http://bmjopen.bmj.com/site/about/guidelines.xhtml

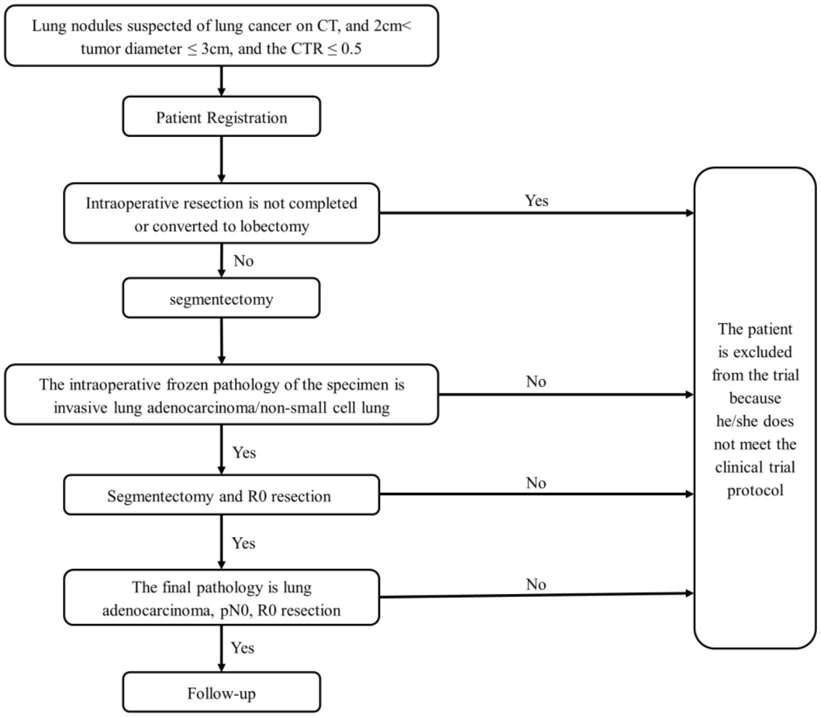

254x190mm (300 x 300 DPI)

筛选号：□□□

肺段切除术治疗 2-3cm 磨玻璃成分为主型浸润性肺癌的单臂、多中心、前瞻性、验证性 III 期临床试验

知 情 同 意 书

For peer review only

方案号：FUSCC2211

版本号：2.0

版本日期：2023 年 01 月 01 日

知情页

【前言】

由于您已经被诊断可能患有非小细胞肺癌，故在此邀请您参加一项临床研究。在您同意参加该项研究之前，请您仔细阅读这份知情同意书，并向您的医生或研究人员提出任何您需要了解的问题，直至得到满意的答复。如果您同意参加本研究，请您签署此知情同意书，并且保存该份经您和研究者双方签字的知情同意书副本。

【研究目的和背景】

本研究的目的是评估肺段切除术对 2-3cm 磨玻璃成分为主型肺癌的疗效。

肺段切除术因其能够保留更多的正常肺组织而越来越受到重视。一项来自日本的单臂研究纳入了长径≤2cm、实性成分占比（consolidation-to-tumor ratio，CTR）≤0.25 周围型肺癌，并对其行亚肺叶切除，结果表明该组患者的术后五年无复发生存率（recurrence-free survival，RFS）达到 99.7%，且没有发生局部复发。而另一项来自日本的随机对照研究则研究了在长径≤2cm、0.5<CTR≤1 周围型肺癌中肺段切除术和肺叶切除术的治疗效果，结果表明肺段切除术的术后生存不劣于肺叶切除术，因此肺段切除术应该是周围型小肺癌的首选治疗方式。来自美国的一项随机对照研究也探究了在不大于 2cm 肺癌中亚肺叶切除术和肺叶切除术的治疗效果，也证实了亚肺叶切除可用于治疗 T1aN0 且肿瘤≤2cm 的肺癌。

然而，目前针对 2<长径≤3cm、CTR≤0.5 肺癌的手术方式还没有定论。一项来自日本的单臂临床研究（JCOG1211）纳入了下列患者：（1）2<长径≤3cm、CTR≤0.5；（2）长径≤2cm、0.25<CTR≤0.5；（3）长径≤2cm、CTR≤0.25 且楔形无法完全切除。只要患者符合上述条件的任意一条，就可纳入该研究，纳入的患者均接受了肺段切除。虽然，该研究纳入了 2<长径≤3cm、CTR≤0.5 肺癌患者，但是这个临床研究还含有相当一部分影像学非浸润的患者，这部分患者的预后极佳，这可能是导致整体预后较好的原因。所以，目前有必要针对 2<长径≤3cm、CTR≤0.5 浸润性肺腺癌的最佳手术方式展开探究。

【研究设计和步骤】

本研究为单臂、多中心、前瞻性、验证性 III 期临床研究。目前针对 2<长径≤3cm、CTR≤0.5 浸润性肺腺癌的患者，临床可以实行肺段切除术，也可以实行肺叶切除术。但肺段切除术，可以保留更多的正常肺组织。本研究纳入的患者将全部接受肺段切除术。如果您符合入选条件并同意参加该项研究的话，我们会记录您术中及术后的病理结果以用于后续分析统计。

您参与的这个研究将包括下列步骤：

➢ **筛选及术前评估期**

如果您选择参加本次临床研究，您的医生或其研究团队成员将首先评价该研究是否适合您。为了确定这一情况，在入组前 2 周内您将进行一次全面的医学检查，包括：

For peer review only - http://bmjopen.bmj.com/site/about/guidelines.xhtml

- 病史： 您的医生会问一些有关您健康的问题以及最近您使用的药物或其他治疗情况。重要的是告诉医生所有您正在使用药物或其他治疗，包括任何您自行购买的药物。
- 体格检查： 您的医生将对您的身体进行检查，包括测量身高、体重、体温、呼吸频率、脉搏以及血压等。您的医生也将对您进行日常生活能力的评估。
- 如果已活检，您的医生将分析您的病理标本，以明确病理结果为非小细胞肺癌
- 影像学检查：您的医生将分析您的胸部 CT、头颅核磁共振、彩超、骨扫描或 PET/CT 等影像学检查结果，以明确您的肺腺癌为临床 T1N0M0，Ia 期。
- 您的医生将通过心电图了解您的心脏情况。
- 常规血液检查： 将用来检查您的血细胞情况、凝血情况、肿瘤标记物癌胚抗原、肾脏或肝脏等器官的工作情况。这些检查大约需要收集 2 大汤匙的血液。
- 尿液检查： 用来排除泌尿系感染和蛋白尿等异常情况。

如果这些检查的结果提示参加该临床研究可能使您的健康受到危险，您的医生将不会建议您进入本次研究，并同您讨论其他的治疗选择。但如果这些检查结果显示您适合该研究，您可以选择参加这一临床研究。

➢ **手术治疗期**

签署知情同意书后，我们将按计划为您进行肺段切除术。在术中，您的医生将记录肿瘤冰冻病理的情况，该检查不会额外增加手术时间和创伤。

➢ **术后随访期**

如果您已入组并顺利接受了肺段切除术，术后 1 个月内，您的医生将记录石蜡病理的信息，包括肿瘤病理类型及淋巴结转移情况。

术后 5 年内，您会接到专业医师的随访电话，询问您疾病相关情况及恢复情况。

【可能的风险与不适】

本研究所有数据均基于临床已有数据，不会对您早成额外的创伤，但肺癌切除手术本身可能存在风险，您的医生将在手术前向您仔细介绍手术和麻醉的相关风险。

【可能的受益】

本次研究的治疗方案并不改变目前的标准临床实践。但您的健康状况和临床资料将有专职研究者进行评估。在试验期间，您也可能不会从本研究中直接获得更多的好处，但是您所提供的宝贵的医学信息将为日后非小细胞性肺癌患者的治疗提供帮助。

【标本采集】

本研究不涉及额外的标本采集，所有检验及检查均为术前常规检查，术中标本的评估也不需要额外采集组织标本

For peer review only - http://bmjopen.bmj.com/site/about/guidelines.xhtml
For peer review only

【肿瘤组织样本和血液样本留存】

如果您选择参加本项研究，为了更好地了解非小细胞肺癌及其治疗方法，研究者将使用您手术后切除的肿瘤组织的一部分样本用于将来与非小细胞肺癌有关的探索性研究。

您的肿瘤组织或标本可能会在相应的实验室或运送至中心实验室长期保存，直至样本全部用完或当时保存条件的极限。

被保存的您的肿瘤组织以及血标本的探索性研究可能会花费几年时间，但如果有成效则可能有助于非小细胞肺癌的早期诊断或更有效的治疗。

【替代治疗】

参加本研究是完全自愿的，如果您选择不参加、或在研究的任何阶段选择退出，不会影响您既定的治疗方案。

【新发现】

在研究期间，若有任何关于疾病或任何关于药物会影响您决定是否继续参加本研究的重要信息，您的医生将及时通知您。试验期间，您可随时了解与其有关的信息资料。

【请您配合的事项】

假使您选择参与本研究，研究期间请密切配合研究者的工作，并请遵守以下事项：

（1） 按时到医院随访；

（2） 请告诉医生试验过程的任何不适，以便医生对症处理；

（3） 您需要在研究过程中向医生或研究者提供有关您的健康资讯，特别是任何有利或不利的改变。

【保密】

您的病情信息在任何时候都会被保密。只有研究医生保留您的基本信息，在研究的其他文件中将使用您的姓名缩写和代码标识。除了您的研究医生外，医院的伦理委员会的代表可能将会查阅您与研究有关的原始医疗资料以确保研究是规范的，数据是真实可靠的。但所有的信息将会保密，本研究结果可能会发表在医学杂志上，但也不会泄露您的身份。

【参加原则】

您参加本研究完全是自愿的。您有权随时退出研究而您的医疗待遇与权益也不会受到影响。您有权了解有关的研究用药可能带来的不良反应。若继续参加研究会对您的健康造成伤害，或您的健康已不适合继续参加试验，或您不能遵守试验方案的要求，您的医生可以终止您的研究而不必得到您的同意。如果您决定退出研究，请您务必与负责试验的医生联系，医生将会安排您回诊一次以作各项指标的评估。

本研究的研究者也可能会出于安全或其它方面的考虑提前中止整个研究或对整个研究做出限制。

For peer review only - http://bmjopen.bmj.com/site/about/guidelines.xhtml

知情同意书签字页

我在充分了解受试者须知的全部内容以及参加本研究可能带来的利弊后，自愿参加本研究，并做出以下申明：

1. 作为受试者，我已阅读了上述受试者须知内容并理解本研究的性质、目的及该药物可能出现的不良反应等信息，我的问题已经得到满意的答复。
2. 我同意在研究中按时就诊随访，并接受与本研究有关的相应检查。我将遵守受试者须知要求，并与研究人员充分合作，如实、客观地向研究人员提供参加本研究前、研究期间和各随访期的健康状况及相关情况；
3. 我亦明白我可以随时退出研究，而此后的治疗并不会因此受到不利影响。我理解研究者有权根据我的情况随时终止研究；
4. 我明白我会收到一份签署过的知情同意书副本；
5. 我同意在此项医学研究中收集、使用和发表我的医学健康数据。
6. 经过充分考虑后，我自愿参加肺段切除术治疗 2-3cm 磨玻璃成分为主型浸润性肺癌的单臂、多中心、前瞻性、验证性 III 期临床试验。
7. 若我有任何关于研究和受试者权益的信息问题时，可以与以下研究人员联系：研究医生_傅方求_，电话_64175590-82500

受试者签名：________________ **日期：**____**年**___**月**___**日**

（仅当受试者不能阅读或签字时需法定监护人签名）

受试者法定监护人签名：____________ **日期：**____**年**___**月**___**日**

我已对上述参加研究的自愿者解释了该项研究的有关细节，并且为他/她提供一份签署过的知情同意书副本。

研究者签名：____________ **日期：**____**年**___**月**___**日**

For peer review only - http://bmjopen.bmj.com/site/about/guidelines.xhtml

Reporting checklist for protocol of a clinical trial.

Based on the SPIRIT guidelines.

Instructions to authors

Complete this checklist by entering the page numbers from your manuscript where readers will find each of the items listed below.

Your article may not currently address all the items on the checklist. Please modify your text to include the missing information. If you are certain that an item does not apply, please write "n/a" and provide a short explanation.

Upload your completed checklist as an extra file when you submit to a journal.

In your methods section, say that you used the SPIRITreporting guidelines, and cite them as:

Chan A-W, Tetzlaff JM, Gøtzsche PC, Altman DG, Mann H, Berlin J, Dickersin K, Hróbjartsson A, Schulz KF, Parulekar WR, Krleža-Jerić K, Laupacis A, Moher D. SPIRIT 2013 Explanation and Elaboration: Guidance for protocols of clinical trials. BMJ. 2013;346:e7586

For peer review only

		Reporting Item	Page Number
**Administrative information**			
Title	#1	Descriptive title identifying the study design, population, interventions, and, if applicable, trial acronym	1
Trial registration	#2a	Trial identifier and registry name. If not yet registered,	2

		name of intended registry	
Trial registration: data set	#2b	All items from the World Health Organization Trial Registration Data Set	n/a
Protocol version	#3	Date and version identifier	n/a
Funding	#4	Sources and types of financial, material, and other support	8
Roles and responsibilities: contributorship	#5a	Names, affiliations, and roles of protocol contributors	8
Roles and responsibilities: sponsor contact information	#5b	Name and contact information for the trial sponsor	1
Roles and responsibilities: sponsor and funder	#5c	Role of study sponsor and funders, if any, in study design; collection, management, analysis, and interpretation of data; writing of the report; and the decision to submit the report for publication, including whether they will have ultimate authority over any of these activities	8
Roles and responsibilities: committees	#5d	Composition, roles, and responsibilities of the coordinating centre, steering committee, endpoint adjudication committee, data management team, and other individuals or groups overseeing the trial, if applicable (see Item 21a for data monitoring committee)	n/a

Introduction

For peer review only

Background and rationale	#6a	Description of research question and justification for undertaking the trial, including summary of relevant studies (published and unpublished) examining benefits and harms for each intervention	2-3
Background and rationale: choice of comparators	#6b	Explanation for choice of comparators	2-3
Objectives	#7	Specific objectives or hypotheses	2-3
Trial design	#8	Description of trial design including type of trial (eg, parallel group, crossover, factorial, single group), allocation ratio, and framework (eg, superiority, equivalence, non-inferiority, exploratory)	2-3
**Methods: Participants, interventions, and outcomes**			
Study setting	#9	Description of study settings (eg, community clinic, academic hospital) and list of countries where data will be collected. Reference to where list of study sites can be obtained	3-4
Eligibility criteria	#10	Inclusion and exclusion criteria for participants. If applicable, eligibility criteria for study centres and individuals who will perform the interventions (eg,	4-5

For peer review only

		surgeons, psychotherapists)	
Interventions: description	#11a	Interventions for each group with sufficient detail to allow replication, including how and when they will be administered	5-6
Interventions: modifications	#11b	Criteria for discontinuing or modifying allocated interventions for a given trial participant (eg, drug dose change in response to harms, participant request, or improving / worsening disease)	5-6
Interventions: adherance	#11c	Strategies to improve adherence to intervention protocols, and any procedures for monitoring adherence (eg, drug tablet return; laboratory tests)	5-6
Interventions: concomitant care	#11d	Relevant concomitant care and interventions that are permitted or prohibited during the trial	5-6
Outcomes	#12	Primary, secondary, and other outcomes, including the specific measurement variable (eg, systolic blood pressure), analysis metric (eg, change from baseline, final value, time to event), method of aggregation (eg, median, proportion), and time point for each outcome. Explanation of the clinical relevance of chosen efficacy and harm outcomes is strongly recommended	4
Participant timeline	#13	Time schedule of enrolment, interventions (including any run-ins and washouts), assessments, and visits for participants. A schematic diagram is highly recommended (see Figure)	3

For peer review only

Sample size	#14	Estimated number of participants needed to achieve study objectives and how it was determined, including clinical and statistical assumptions supporting any sample size calculations	7
Recruitment	#15	Strategies for achieving adequate participant enrolment to reach target sample size	7
**Methods: Assignment of interventions (for controlled trials)**			
Allocation: sequence generation	#16a	Method of generating the allocation sequence (eg, computer-generated random numbers), and list of any factors for stratification. To reduce predictability of a random sequence, details of any planned restriction (eg, blocking) should be provided in a separate document that is unavailable to those who enrol participants or assign interventions	n/a
Allocation concealment mechanism	#16b	Mechanism of implementing the allocation sequence (eg, central telephone; sequentially numbered, opaque, sealed envelopes), describing any steps to conceal the sequence until interventions are assigned	n/a
Allocation: implementation	#16c	Who will generate the allocation sequence, who will enrol participants, and who will assign participants to interventions	n/a

For peer review only

Blinding (masking)	#17a	Who will be blinded after assignment to interventions (eg, trial participants, care providers, outcome assessors, data analysts), and how	n/a
Blinding (masking): emergency unblinding	#17b	If blinded, circumstances under which unblinding is permissible, and procedure for revealing a participant's allocated intervention during the trial	n/a
**Methods: Data collection, management, and analysis**			
Data collection plan	#18a	Plans for assessment and collection of outcome, baseline, and other trial data, including any related processes to promote data quality (eg, duplicate measurements, training of assessors) and a description of study instruments (eg, questionnaires, laboratory tests) along with their reliability and validity, if known. Reference to where data collection forms can be found, if not in the protocol	7
Data collection plan: retention	#18b	Plans to promote participant retention and complete follow-up, including list of any outcome data to be collected for participants who discontinue or deviate from intervention protocols	7
Data management	#19	Plans for data entry, coding, security, and storage, including any related processes to promote data quality (eg, double data entry; range checks for data values).	7

For peer review only

		Reference to where details of data management procedures can be found, if not in the protocol	
Statistics: outcomes	#20a	Statistical methods for analysing primary and secondary outcomes. Reference to where other details of the statistical analysis plan can be found, if not in the protocol	7
Statistics: additional analyses	#20b	Methods for any additional analyses (eg, subgroup and adjusted analyses)	7
Statistics: analysis population and missing data	#20c	Definition of analysis population relating to protocol non-adherence (eg, as randomised analysis), and any statistical methods to handle missing data (eg, multiple imputation)	7
**Methods: Monitoring**			
Data monitoring: formal committee	#21a	Composition of data monitoring committee (DMC); summary of its role and reporting structure; statement of whether it is independent from the sponsor and competing interests; and reference to where further details about its charter can be found, if not in the protocol. Alternatively, an explanation of why a DMC is not needed	7
Data monitoring: interim analysis	#21b	Description of any interim analyses and stopping guidelines, including who will have access to these interim results and make the final decision to terminate the trial	7
Harms	#22	Plans for collecting, assessing, reporting, and managing solicited and spontaneously reported adverse events and other unintended effects of trial interventions or trial	n/a

For peer review only

		conduct	
Auditing	#23	Frequency and procedures for auditing trial conduct, if any, and whether the process will be independent from investigators and the sponsor	n/a
**Ethics and dissemination**			
Research ethics approval	#24	Plans for seeking research ethics committee / institutional review board (REC / IRB) approval	8
Protocol amendments	#25	Plans for communicating important protocol modifications (eg, changes to eligibility criteria, outcomes, analyses) to relevant parties (eg, investigators, REC / IRBs, trial participants, trial registries, journals, regulators)	n/a
Consent or assent	#26a	Who will obtain informed consent or assent from potential trial participants or authorised surrogates, and how (see Item 32)	8
Consent or assent: ancillary studies	#26b	Additional consent provisions for collection and use of participant data and biological specimens in ancillary studies, if applicable	n/a
Confidentiality	#27	How personal information about potential and enrolled participants will be collected, shared, and maintained in order to protect confidentiality before, during, and after the trial	8
Declaration of	#28	Financial and other competing interests for principal	9

For peer review only

interests		investigators for the overall trial and each study site	
Data access	#29	Statement of who will have access to the final trial dataset, and disclosure of contractual agreements that limit such access for investigators	7-8
Ancillary and post trial care	#30	Provisions, if any, for ancillary and post-trial care, and for compensation to those who suffer harm from trial participation	n/a
Dissemination policy: trial results	#31a	Plans for investigators and sponsor to communicate trial results to participants, healthcare professionals, the public, and other relevant groups (eg, via publication, reporting in results databases, or other data sharing arrangements), including any publication restrictions	7
Dissemination policy: authorship	#31b	Authorship eligibility guidelines and any intended use of professional writers	n/a
Dissemination policy: reproducible research	#31c	Plans, if any, for granting public access to the full protocol, participant-level dataset, and statistical code	n/a
**Appendices**			
Informed consent materials	#32	Model consent form and other related documentation given to participants and authorised surrogates	n/a
Biological specimens	#33	Plans for collection, laboratory evaluation, and storage of biological specimens for genetic or molecular analysis in the current trial and for future use in ancillary studies, if applicable	n/a

For peer review only

The SPIRIT Explanation and Elaboration paper is distributed under the terms of the Creative Commons Attribution License CC-BY-NC. This checklist was completed on 30. March 2024 using https://www.goodreports.org/, a tool made by the EQUATOR Network in collaboration with Penelope.ai

For peer review only
